# Genetic stability of a recombinant adenovirus vaccine vector seed library expressing human papillomavirus type 16 E6 and E7 proteins

**DOI:** 10.3892/etm.2015.2268

**Published:** 2015-02-05

**Authors:** JIE WU, KE-DA CHEN, MENG GAO, GANG CHEN, SU-FENG JIN, FANG-CHENG ZHUANG, XIAO-HONG WU, YUN-SHUI JIANG, JIAN-BO LI

**Affiliations:** Institute of Viral Diseases, Zhejiang Academy of Medical Sciences, Hangzhou, Zhejiang 310053, P.R. China

**Keywords:** human papillomavirus type 16, adenovirus vector, seed bank, genetic stability

## Abstract

The aim of the present study was to understand the genetic stability of a master seed bank (MSB) and a working seed bank (WSB) of an adenovirus vector vaccine expressing the human papillomavirus (HPV) type 16 E6 and E7 fusion proteins (Ad-HPV16E6E7). Microscopic examination and viral infectious efficacy were used to measure the infectious titers of the Ad-HPV16E6E7 MSB and WSB. Polymerase chain reaction was used to analyze the stability of the Ad-HPV16E6E7 target gene insertion, while western blot analysis and immunofluorescence were used to assess the expression levels of the Ad-HPV16E6E7 target protein. A C57BL/6 mouse TC-1 tumor cell growth inhibition model was used to evaluate the biological effect of Ad-HPV16E6E7 administration. The infectious titers of the Ad-HPV16E6E7 MSB and WSB were 6.31×10^9^ IU/ml and 3.0×10^9^ IU/ml, respectively. In addition, the expression levels of the inserted target genes and target proteins were found to be stable. In the mouse TC-1 tumor inhibition analysis, when the virus titers of the Ad-HPV16E6E7 MSB and WSB were 10^9^ IU/ml, the tumor inhibition rate was 100%, which was significantly different when compared with the control group (χ^2^MSB=20.00 and χ^2^WSB=20.00; P<0.01). Therefore, the Ad-HPV16E6E7 vaccine seed bank is genetically stable and meets the requirements for vaccine development.

## Introduction

Human papillomavirus (HPV) infection is closely associated with cervical cancer. Numerous studies from around the world have reported that >90% of cervical cancer and precancerous lesions contain HPV DNA (1,2). Among these cases, high-risk HPV type 16 infection is the most important factor in tumorigenesis (3), and subsequently the first choice target for vaccine development. HPV type 16 E6 and E7 proteins are continuously expressed in cervical cancer and precancerous lesions, and are a completely exogenous viral antigen. Therefore, these proteins are an ideal target antigen for a HPV treatment vaccine (4).

A replication-defective recombinant adenovirus has the advantage of having a wide range of hosts, without integrating into the host cells. Furthermore, large genes (up to 37 kb) may be inserted into adenovirus vectors, and the vector can be used to express multiple genes at the same time at high expression levels. The expressed antigen is modified and presented in the host cells, which subsequently produces strong humoral and cell immunity. All these characteristics make the adenovirus a suitable vector for tumor vaccines.

Research into a HPV type 16 vaccine using an adenovirus as the vector has entered the development stage (5). Furthermore, in China, the early stage construction and the first-stage immunity evaluation of a HPV type 16 E6/E7 recombinant adenovirus vector vaccine, developed by the Houwen Tian research group from the Virus Institute at the Chinese Center for Disease Control and Prevention (Beijing, China), have been completed (6). According to the technology agreement, the present research group constructed the master seed bank (MSB) and working seed bank (WSB) required for expressing the HPV type 16 E6/E7 vaccine. In accordance with the management procedure requirements for toxic bacteria species used for biological production from the State Food and Drug Administration, as previously described (7,8), the replication ability of the recombinant adenovirus vector vaccine seed libraries (MSB and WSB), the genetic stability of the inserted target genes and the expression of the target proteins and their biological effects were studied. The present study reports these results.

## Materials and methods

### Materials

A recombinant adenovirus rAd5HPV16SmE7E6 strain was provided by the Viral Diseases Prevention and Control Institute at the Chinese Center for Disease Control and Prevention, according to a technology transfer agreement (4).

HEK293 cells were purchased from the American Type Culture Collection (ATCC; CRL-1573; Manassas, VA, USA). HEK293-EcRShh (adv5) cells were verified by the Chinese Academy of Food and Drug Testing and were passaged to establish the cell library.

### Major reagents

Dulbecco’s modified Eagle’s medium (DMEM) was purchased from Gibco Life Technologies (Beijing, China) and viral genomic DNA/RNA extraction kits were purchased from Tiangen Biotech (Beijing) Co., Ltd. (DP-315; Beijing, China). A Trans2K Plus II DNA Marker was purchased from Beijing Quanshijin Biology Technology Co., Ltd. (Beijing, China). A primary antibody against HPV type 16 E7 (sc-6981) was purchased from Santa Cruz Biotechnology, Inc. (Dallas, TX, USA), while a secondary horseradish peroxidase (HRP)-conjugated goat-anti-mouse IgG antibody was purchased from Beijing Zhongshan Jinqiao Biotechnology Co., Ltd. (Beijing, China). Polymerase chain reaction (PCR) primers were synthesized by Shanghai ShengGong Biological Engineering Technology Co., Ltd. (Shanghai, China). Female C57BL/6 mice of specific-pathogen free grade (age, 6–8 weeks) were purchased from Shanghai Xipuer-Bikai Experimental Animal Co., Ltd. (Shanghai, China) and the TC-1 tumor cell line was purchased from the ATCC (CRL-2785).

### Cell passage of the recombinant adenovirus expressing HPV E6/E7 and infectious viral titer measurements

A recombinant adenovirus expressing HPV type 16 E6/E7 proteins was passaged continuously in HEK293 cells to passages 14 and 15. The infected cells were subsequently harvested, frozen and thawed three times. The harvested liquid was serially diluted, and dilutions ranging between 10^−3^ and 10^−10^ were used to infect the seeded cells in 96-well plates. After 48 h of incubation, the cell morphology was observed, and the infected cells appeared and were counted under a microscope (Olympus CKX41; Olympus, Tokyo, Japan) to calculate the infectious titers.

### Verification of the inserted target genes

For PCR, 1 μl samples were used. In total, five samples were analyzed, including the negative control (adenovirus without the E6/E7 genes, subsequently referred to as the empty adenovirus), the positive control (rAd5HPV16SmE6E7), the two experimental samples (Ad-HPV16E6E7 MSB and WSB) and a blank control (reagents without real samples).

The target gene, HPV16 E6/E7, was ~800 bp in length, and the amplification primers were as follows: mCMVup, CAGTCTTCGGTCTGACCACCG; and pE6dn, GGC CGAATTCATCACAGCTGGGTCTCTCTTC. If a sample showed a band of ~800 bp, the target gene was considered to have successfully been inserted. The PCR amplification program was as follows: Predenaturation at 94°C for 50 sec; followed by 35 cycles of denaturation at 94°C for 30 sec, annealing at 51°C for 30 sec and extension at 72°C for 1 min; and a final extension at 72°C for 10 min. Following PCR, a 5-μl sample of the PCR product was analyzed on 1.0% agarose gel electrophoresis to verify the product size.

### Western blot analysis detection of the Ad-HPV16E6E7 target protein

HEK293 cells were grown in 25-cm^2^ cell culture flasks until 90% confluence was reached. The empty adenovirus, Ad-HPV16E6E7 MSB and WSB were used to infect the cells. The viruses were incubated at 37°C for 1–2 h, and the DMEM were added. The cells were incubated at 37°C in 5% CO_2_ for 24–48 h until all the cells were infected. The supernatant was discarded, and the cells were washed with phosphate-buffered saline (PBS) twice and then harvested with scrapers. Next, 50 μl 2X SDS loading buffer was added to each sample and the samples were boiled at 100°C for 3 min. Samples of 20 μl were loaded onto the gel, analyzed by 10% SDS-PAGE and transferred to a nitrocellulose membrane. An anti-HPV type 16 E7 mouse monoclonal antibody (1:1,000) was used as the primary antibody, while a HRP-conjugated goat anti mouse IgG (1:2,000) was used as the secondary antibody. The immunoblots were developed using an ECL chemiluminescence detection system (Thermo Fisher Scientific, Waltham, MA, USA) and visualized using a Kodak BIOMAX Light Film (Sigma-Aldrich, St. Louis, MO, USA).

### Immunofluorescence detection of the Ad-HPV16E6E7 target protein

When the HEK293 cells reached 80–90% confluence, the cells were infected with the empty virus, Ad-HPV16E6E7 MSB or WSB. The cells were incubated at 37°C for 1–2 h, and the media were added. The cells were incubated further at 37°C for 36–48 h, and subsequently digested with trypsin and centrifuged at 1,000 rpm for 5 min (centrifugal radius of 6.5 cm). The supernatants were discarded and the pellets were washed with PBS three times. The cells were put on slides, blocked with blocking solution (methyl acetone; 1:1) for 10 min and dried. The antibodies were diluted with PBS, and a 5–10-μl sample of the HPV type 16 E7 monoclonal primary antibody was incubated with the samples at room temperature for 2 h. Moisture was maintained to prevent antibody evaporation. The samples were gently washed with K^+^-free PBS three times, each time for 2–3 min. An immunofluorescence-labeled secondary antibody (aMo IgG; cat. no. ZF-0312; 1:100; ZSGB-Bio, Beijing, China) was added and incubated for 2 h at room temperature (25°C). The sample was stained with Evans blue (cat. no. ab120869; Abcam, Sangon Biotec Co., Ltd., Shanghai, China) for 10 min and then observed under a fluorescence microscope (Leica DM13000B; Leica Microsystems GmbH, Wetzlar, Germany).

### Mouse tumor growth inhibition assay

In total, 50 C57BL/6 mice were used in the study. Each mouse received 1×10^4^ TC-1 tumor cells by an injection into the groin to induce tumor growth. After 24 h (labeled as day 0), the mice were divided randomly into the PBS control group, the empty adenovirus control group (2.85×10^9^ IU/ml), the MSB group (6.31×10^9^ IU/ml), the WSB group (3.00×10^9^ IU/ml) and the S090304B recombinant adenovirus group (3.00×10^7^ IU/ml). There were 10 mice in each group. All the mice received intramuscular administration, and the injection volume was 100 μl. Tumor growth was observed at day 0, 7, 14, 21, 28, 35 and 42. S090304B was the test product developed by the Viral Diseases Institute at Zhejiang Academy of Medical Sciences (Hangzhou, China). The aim of this experiment was to analyze the effect of the vaccine on mouse TC-1 tumor cell growth inhibition. The present study was approved by the ethics committee of Zhejiang Academy of Medical Sciences (Hangzhou, China).

### Statistical analysis

Tumor inhibition rates among the groups were compared using the χ^2^ test, where P<0.05 was considered to indicate a statistically significant difference.

## Results

### Characteristics of the recombinant adenoviruses expressing HPV E6/E7 in HEK293 cells

Recombinant adenoviruses expressing the HPV type 16 E6/E7 MSB and WSB were continuously passaged and used to infect HEK293 cells. The infection time was stable and varied between 42 and 48 h. Cell morphology changed from attached polygonal spindle shapes to round particles that gradually detached, showing evident viral plaques. The infected cells exhibited stable morphological changes, and the viruses were harvested. The MSB infectious titer was 6.31×10^9^ IU/ml, while the WSB infectious titer was 3.0×10^9^ IU/ml, which met the seed library titer requirement.

### PCR analysis of the inserted target HPV type 16 E6/E7 gene

**Figure 2:**
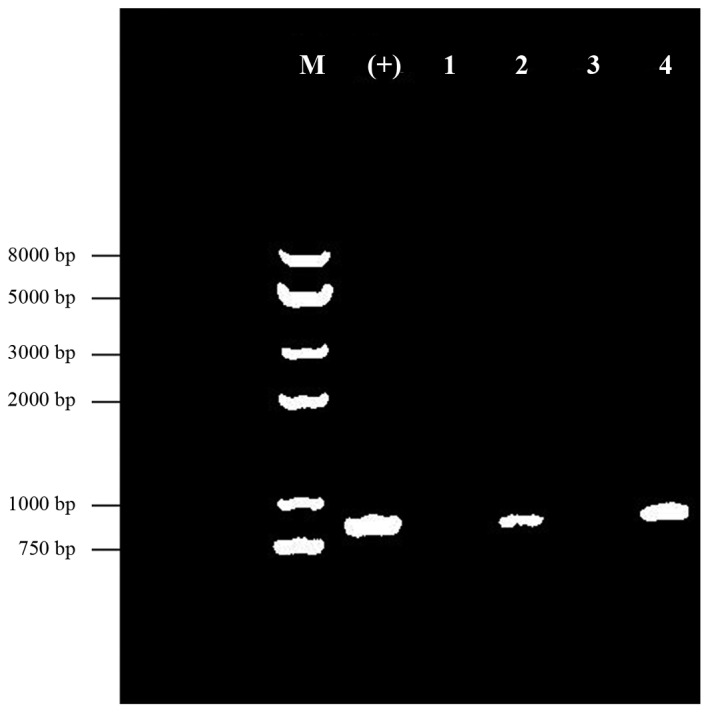
Polymerase chain reaction (PCR) analysis of the master seed bank (MSB) and working seed bank (WSB) of the recombinant adenovirus expressing human papillomavirus type 16 E6/E7 (PCR primers: mCMVup, CAGTCTTCGGTCTGACCACCG and pE6dn, GGCCGAATTCATCACAGCTGGGTCTCTCTTC). Lanes: M, DNA marker; (+), positive control; 1, negative control; 2, MSB sample; 3, blank control; 4, WSB sample.

The 771-bp, codon-optimized HPV type 16 E6/E7 gene was inserted into the adenovirus. Agarose gel electrophoresis analysis revealed that the negative control and blank control did not yield any gene amplification bands. The amplification products using the mCMVup and pE6dn primer set showed specific bands of ~800 bp in length. Thus, the results demonstrated specific amplification of the bands (Fig. 2) in the MSB and WSB samples.

### Western blot analysis of HPV type 16 E6/E7 expression

**Figure 3:**
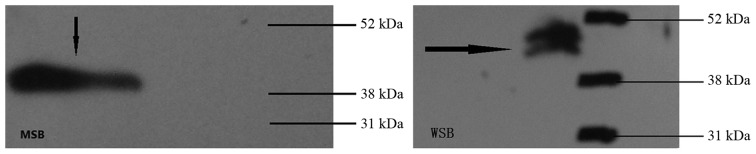
Western blot analysis of the recombinant adenovirus expressing human papillomavirus type 16 E6/E7 proteins, MSB and WSB fusion gene expression products. MSB, master seed bank; WSB, working seed bank. Arrows indicate specific proteins.

Results from western blot analysis are shown in Fig. 3. The MSB and WSB showed specific bands at a molecular weight of 40,000 Da, indicating that the MSB and WSB expressed the HPV E6/E7 fusion proteins. However, this size was larger than the predicted protein size of 35,000 Da, which may be due to protein phosphorylation. This band was observed due to the specific binding between the monoclonal E7 antibody and the protein of interest. Thus, the band was not detected in the empty adenovirus sample.

### Immunofluorescence analysis of HPV type 16 E6/E7 expression

**Figure 4:**
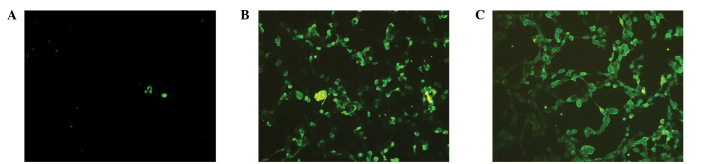
Immunofluorescence analysis of the master seed bank (MSB) and working seed bank (WSB) target gene expression using a specific E7 antibody (magnification, ×100). (A) Ad5 (empty virus vector). (B) Fluorescence due to MSB products binding to the specific E7 antibody. (C) Fluorescence of WSB products binding to the specific E7 antibody.

Immunofluorescence analysis is shown in Fig. 4. The MSB and WSB showed specific binding, as detected by immunofluorescence. No recombinant HPV E6/E7 gene or specific immune reaction was observed in the empty adenovirus sample; therefore, there was no immunofluorescence activity indicating specific binding.

### Mouse tumor cell growth inhibition analysis

**Figure 5:**
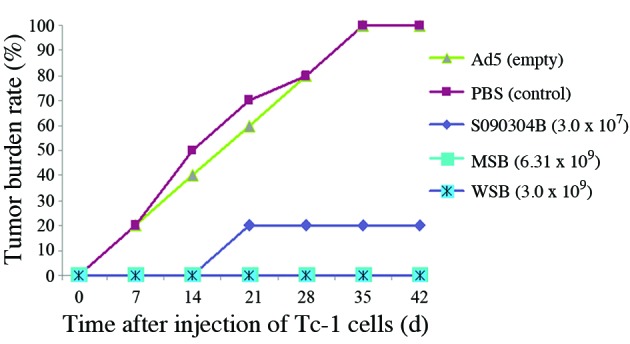
Inhibitory effects of the recombinant adenovirus expressing the human papillomavirus type 16 E6/E7 MSB and WSB on TC-1 tumor cells. PBS, phosphate-buffered saline; MSB, master seed bank; WSB, working seed bank.

Inhibitory effects on mouse tumor cell growth are shown in Fig. 5. Tumor formation was slow in the mice injected with TC-1 tumor cells in the PBS and empty adenovirus control groups. Tumors formed in two mice on day 7 (2/10), in five mice on day 14 (5/10) and in all 10 mice on day 35 (10/10), which was a different result from that of previous experiments in which the control group showed 100% tumor formation in 14–21 days. The number of tumors formed in the mice in the MSB and WSB groups on day 42 was zero; thus, the tumor inhibition rate was 100%. The percentage of tumor formation in the mice in the S090304B group was 20%, while the percentage of tumor formation in the mice from the control group was 100%. The differences between the MSB, WSB and S09034B groups when compared with the control group were statistically significant (χ^2^=20.00, 20.00 and 13.33, respectively; P<0.01). In the S090304B group, two mice started to form tumors on day 21, and the tumor diameters had reached 1.0–1.5 cm by day 42. Therefore, these tumors were smaller compared with those of the mice in the control group on day 42, which had diameters ranging between 2.5 and 3.5 cm.

## Discussion

As vectors, adenoviruses have played an important role in various fields, including gene therapy, live vector vaccine development and *in vitro* gene transfection. Adenovirus vectors have been used in prostate cancer treatment (9). In addition, the recombinant human adenovirus 5 (H101) has been approved as a clinical drug for head and neck cancer treatment in China (10). Their application has produced substantial evidence for the safety and efficacy of adenoviruses used as vectors. Previous research formed the basis for the long-term aim of the present study, which was to treat cervical intraepithelial neoplasia or precancerous lesions using recombinant adenoviruses expressing HPV type 16 E6/E7 (6).

The requirement of a seed library for recombinant adenoviruses used as biological products has been clearly defined (8). As a gene recombination product, the adenovirus vaccine MSB and WSB must assure the stability of the inserted target genes and their expression levels (11), as well as the biological effects of the corresponding proteins. The present study used these indicators to assess adenovirus vaccine seed libraries. The recombinant adenovirus HPV type 16 E6/E7 MSB and WSB were found to have high titers, correct target gene band sizes, stable target protein expression and suitable biological effects in a mouse TC-1 tumor cell growth inhibition assay. These results indicated that MSB and WSB were genetically stable and were able to meet the vaccine seed requirements.

The mouse TC-1 tumor inhibition assay demonstrated that when the titer of recombinant adenovirus was 10^9^ IU/ml, the tumor formation rate was zero. However, when the titer of the S090304B recombinant adenovirus was 10^7^ IU/ml, the tumor formation rate was 20%, indicating that the titer of the adenovirus vector vaccine may affect tumor formation. Theoretically, the production of a recombinant adenovirus vaccine with a 10^9^ IU/ml titer may be highly effective in clinical application. However, obtaining a recombinant adenovirus with such a high titer is impossible in cell culture, which makes it difficult to use it as a product. Nevertheless, the recombinant adenovirus can meet the requirement of a seed library.

However, the results of the present study are limited, since only the stability of the seed library was investigated. According to the requirements of recombinant adenovirus vaccine research, further study is required to investigate the genetic stability of the WSB passaged products, including the vaccine products and the products resulting from six passages of adenovirus vaccine products, to assure the safety and efficacy for human vaccines.

An adenovirus vector vaccine has a number of advantages; however, further improvements are required. The major disadvantage of this type of vaccine is that the original host-background neutralizing antibodies produced following adenovirus infections can reduce the efficacy of the vaccine. In addition, the antiviral antibody produced after the immunization can inhibit reimmunization; therefore, the effects of immunization may be improved through using combined immunization (12). The present study revealed that the Ad-HPV16 E6E7 vaccine seed bank (MSB; WSB) was genetically stable and meets the requirements for vaccine development.

## Figures and Tables

**Figure 1:**
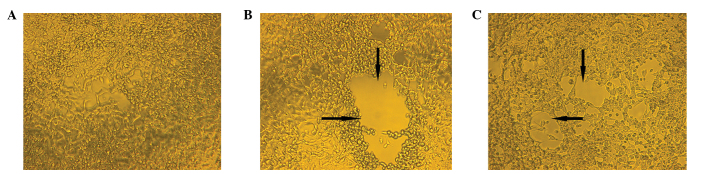
Replication of the recombinant adenovirus expressing human papillomavirus type 16 E6/E7 master seed bank (MSB) and working seed bank (WSB) in (A) normal HEK293 cells, (B) viral plaques of HEK293 cells infected with MSB and (C) viral plaques of HEK293 cells infected with WSB (magnification, ×100) observed under an Olympus CKX41 10X microscope.
